# 549. Application of Punch Grafting for Reconstruction of Glabrous Skin Defects

**DOI:** 10.1093/jbcr/irag033.246

**Published:** 2026-04-06

**Authors:** Chinenye O Ogbonnah, Brittany Lively, Amanda Seymour, Brent Egeland

**Affiliations:** University of Texas at Austin Dell Medical School, Austin, TX; Texas A&M University College of Medicine, Bryan, TX; University of Texas at Austin Dell Medical School, Austin, TX; University of Texas at Austin Dell Medical School, Austin, TX

## Abstract

**Introduction:**

Burn reconstruction of plantar and volar surfaces remains particularly challenging due to the functional and biomechanical demands of glabrous skin. Conventional grafting with hair-bearing donor tissue often leads to hyperkeratosis, scarring, and graft breakdown. The objective of this study was to evaluate clinical outcomes of glabrous punch grafting, a novel technique designed to provide durable coverage while minimizing donor-site morbidity.

**Methods:**

A retrospective review was performed at a comprehensive burn center from 2022-2024. Patients undergoing glabrous punch grafting for plantar or volar defects following burns, trauma, or oncologic resection were included. Six patients met inclusion criteria. Primary outcomes were graft take, infection, and length of stay. Secondary outcomes included number of operations, donor-site morbidity, and functional/aesthetic results. The procedure involved harvesting 4-5 mm punch grafts from adjacent glabrous tissue and implanting them in a grid-like pattern into a prepared wound bed. Similar in principle to Meek micrografting, this approach increases the effective surface area that can be covered from limited donor tissue while preserving 'like replaces like' principle of burn reconstruction.

**Results:**

Six patients underwent glabrous punch grafting (mean age 57.0 ± 6.4 years). Mechanisms of injury included burns (n = 4, 66.7%), crush injury (n = 1, 16.7%), and oncologic resection (n = 1, 16.7%). Reconstructions primarily involved the plantar foot (n = 5, 83.3%) with one p hand case (n = 1, 16.7%). Mean TBSA was 15.8 ± 27.7% (median 4.2%, range 2.5-52.0). Mean follow-up was 24.8 ± 8.9 months. Patients underwent a mean of 10.2 ± 15.0 operations, with mean hospital length of stay of 36.5 ± 30.0 days. Estimated wound area averaged 130.3 ± 108.7 cm^2^ (median 85, range 20-286). Early outcomes were favorable with graft take at post-operative day 5. Long-term graft take was excellent across all patients, with durable incorporation and no graft failures. Color match and texture were satisfactory, donor sites healed with negligible scarring, and no infections or major complications occurred.

**Conclusions:**

Glabrous punch grafting is a safe and reproducible option for reconstruction of plantar and volar wounds. This technique preserves the structural and sensory qualities of glabrous skin, reduces donor morbidity, and offers a practical alternative where traditional grafting methods are limited. These findings suggest potential impact on reconstructive strategies in burn care, and further studies in larger cohorts are warranted to validate long-term function and durability.

**Applicability of Research to Practice:**

There is no defined solution for coverage of glabrous skin. This technique offers a practical, reproducible option that replaces “like with like,” increases surface area coverage, and minimizes donor-site morbidity, making it a strong candidate for incorporation into everyday burn practice.

**Funding for the study:**

N/A.

